# The Effect of Geometric Parameters on Flow and Heat Transfer Characteristics of a Double-Layer Microchannel Heat Sink for High-Power Diode Laser

**DOI:** 10.3390/mi13122072

**Published:** 2022-11-25

**Authors:** Yiwei Gao, Junchao Wang, Mingxuan Cao, Luhao Zang, Hao Liu, Matthew M. F. Yuen, Xiaolei Bai, Ying Wang

**Affiliations:** 1 Department of Intelligent Manufacturing, Wuyi University, Jiangmen 529020, China; 2 Key & Core Technology Innovation Institute of The Greater Bay Area, Guangzhou 510535, China; 3 Department of Mechanical Engineering, Hong Kong University of Science and Technology, Hong Kong 999077, China; 4 School of Physical Science and Technology, Inner Mongolia University, Inner Mongolia Autonomous Region, Hohhot 010021, China

**Keywords:** double-layer U-shaped microchannel heat sink, heat transfer, pressure drop, thermal resistance, pumping power

## Abstract

The effect of the geometric parameters on the flow and heat transfer characteristics of a double-layer U-shape microchannel heat sink (DL-MCHS) for a high-power diode laser was investigated in this work. FLUENT 19.2 based on the finite volume method was employed to analyze the flow and heat transfer performance of DL-MCHS. A single variable approach was used to fully research the impact of different parameters (the number of channels, the channel cross-sectional shape, and the aspect ratio) on the temperature distribution, pressure drop, and thermal resistance of the DL-MCHS. The rectangular DL-MCHS heat transfer performance and pressure drop significantly increased with the rise in the channel’s aspect ratio due to there being a larger wet perimeter and convective heat transfer area. By comparing the thermal resistance of the DL-MCHS at the same power consumption, it was found that the rectangular DL-MCHS with an aspect ratio in the range of 5.1180–6.389 had the best overall performance. With the same cross-sectional area and hydraulic diameter (*A_C_* = 0.36 mm, *D_h_* = 0.417 mm), the thermal resistance of the trapezoidal microchannel heat sink was 32.14% and 42.42% lower than that of the triangular and rectangular ones, respectively, under the condition that the pumping power (*W_pp_*) was 0.2 W. Additionally, the thermal resistance was reduced with the increment of the number of channels inside the DL-MCHS, but this would induce an increased pressure drop. Thus, the channel number has an optimal range, which is between 50 and 80 for the heat sinks in this study. Our study served as a simulation foundation for the semiconductor laser double-layer U-shaped MCHS optimization method using geometric parameters.

## 1. Introduction

Due to the shrinking size of the electronic equipment and the increased power density, the heat generation rate of high-power semiconductor chips has increased at an unprecedented rate in the recent years [1,2,3]. The high-power laser diode (HPLD), which serves as the heart of the semiconductor lasers, produces a lot of heat when it is operating [4,5]. In order to achieve a sufficient heat dissipation capacity, the microchannel heat sink (MCHS) had become the most commonly used heat dissipation structure of the LD [6]. The MCHS incorporates numerous parallel microchannels with widths ranging from 10 to 1000 mm, which can compress the thickness of the thermal boundary of the coolant and widen the liquid–solid interface.

The single-layered microchannel heat sink (SL-MCHS) as an innovative device was developed by Tuckerman and Pease [7] in 1981. In subsequent years, significant research work had been carried out in the microfluidics field to study and improve the thermal performance and fluid flow behavior of the channels by altering the aspect ratio of the channels, the cross-sectional shape of the channel, etc. [8,9,10,11,12,13,14,15,16,17,18]. Regarding the aspect ratio, Sahar et al. [19] proved that the thermal resistance decreased at a constant pressure drop and increased at a constant flow rate as the aspect ratio increased. In a study conducted by Wang et al. [20], the microchannels with higher aspect ratios had a lower thermal resistance, but a higher pressure drop, and the ideal aspect ratio was between 8.9 and 11.4. In terms of the shape of the section optimization, Kewalramani et al. [21] studied trapezoidal microchannels under two boundary conditions. The Poiseuille number and the Nussle number increased with an increasing side angle and aspect ratio. The trapezoidal microchannels were found to have better performance when they were compared to the rectangular ones. Salah et al. [22] found that the hydraulic properties of the microchannels were not affected by the size when the channel spacing was reduced from 50 μm to 0.5 μm.

Allowing more coolant to pass through the channels is one method of reducing the non-uniform temperature distributions. In order to achieve this objective, further studies concerning the heat transfer characteristics of the DL-MCHS are reported, and the results proved that the DL-MCHS had superior thermal performance over a single-layered one [8]. The DL-MCHS was first proposed by Vafai and Zhu [23], and in comparison with the conventional SL-MCHS, the DL-MCHS performs much better in terms of temperature rise and pressure drop. According to Hung et al. [24], the DL-MCHS always exhibited a lower thermal resistance than the SL-MCHS did under the same *W_pp_*. Wong et al. [25] demonstrated that the DL-MCHS with a parallel flow had a better thermal performance when it was compared to the DL-MCHS with a counter flow.

Due to the limitation of the structure of the laser beam combination, the MCHS of the LD must be made into a special double-layer U-shaped structure. The MCHS with such a structure has been widely used in the laser industry. However, the effect of each of the mentioned geometric parameters on the thermal performance of the double-layer U-shaped MCHS has not been thoroughly explored so far. On the other hand, there have been numerous experiments and numerical investigations of the influence of the geometric parameters on its performance, but certain issues remain unsolved. For instance, Chen et al. [26] and Gunnasegaran et al. [27] had examined the effect of different geometric features on the fluid flow and heat transfer in the MCHS. However, their conclusions are in contradiction with one another.

In this paper, the effects of parameters such as the number of channels, the channel cross-sectional shape, and the channel aspect ratio on the temperature distribution, pressure drop, and thermal resistance of the MCHS were investigated. The rectangular DL-MCHS’s heat transfer performance and pressure drop significantly increased with a higher aspect ratio due to there being a larger wet perimeter and convective heat transfer area. According to the thermal resistance of the DL-MCHS at the same power consumption rates, the rectangular DL-MCHS with an aspect ratio in the range of 5.1180–6.389 had the best overall performance. With the same cross-sectional area and hydraulic diameter (*A_C_* = 0.36 mm, *D_h_* = 0.417 mm), the thermal resistance of the trapezoidal MCHS was 32.14% and 42.42% lower than that of the triangular and rectangular ones, respectively, under the condition that the *W_pp_* was 0.2 W. Additionally, the thermal resistance reduced with the increment of the number of channels inside the DL-MCHS, but this would induce an increased pressure drop. Thus, the channel number has an optimal range, which was between 50 and 80 for the heat sinks in this study. Our study served as a simulation basis for the semiconductor laser double-layer U-shaped MCHS optimization method.

## 2. Numerical Model Construct

The parameters of the model we built are based on the commercial microchannel heat sink produced by the Rogers Corporation. The number of microchannels was usually 10–14, the channel diameter was 0.2–0.6 mm, and the structure of the microchannel heat sink was symmetrical from left to right. Based on Diahua Wu’s research [28], since the heat spreading along a laser cavity to the rear side is within a finite length, only the part of the microchannel heat sink that involved in heat convection should be considered in the thermal modeling, while the other part of the microchannel heat sink structure can be ignored to simplify the physical model.

**Figure 1:**
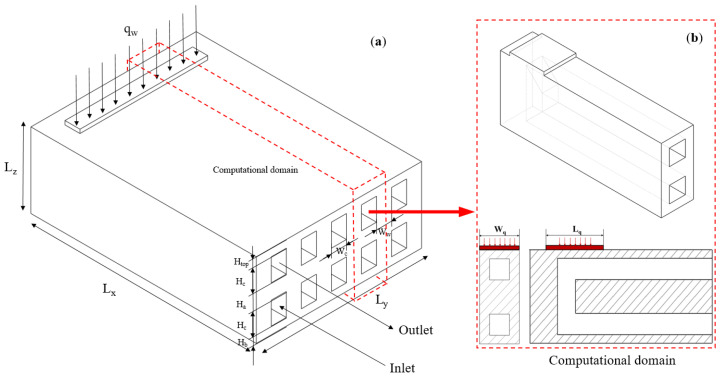
Schematic diagram of microchannel heat sink geometry (**a**) and computational domain (**b**).

The simplified structure of the designed double-layer U-shaped MCHS is shown in Figure 1. The microchannel structure was made of oxygen-free copper with a high thermal conductivity, and deionized water served as the cooling medium. Table 1 listed the physical and thermal characteristics of these materials. The LD bar chips needed to be soldered to the front edge of the microchannel heat sink, and the heat that was generated by the chip was conducted by the solid part of the microchannel heat sink to the fluid–solid interface of the microchannel, which in turn was carried away by the flowing refrigerant liquid. The specific parameters of its external dimensions are shown in Table 2. The width, height, and length of the heat sink, respectively, are represented by *W*, *H*, and *L*. The channel width and height are expressed by *W_C_* and *H_C_*. Specifically, *Ha* represents the distance between the upper and lower channels. *W_W_* is the thickness of the sidewall, while *H_top_* and *H_b_* are the thickness of the top wall and heat sink bottom wall, respectively.

**Figure 2:**
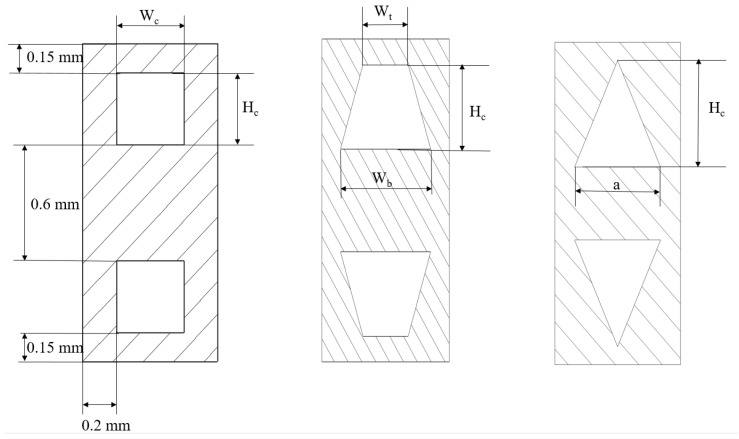
Different cross-sectional shapes of microchannels and their dimensional parameters.

All of the channels in the DL-MCHS had a rectangular cross-section and the same geometric dimensions. Due to the symmetrical nature of the DL-MCHS physical model, a symmetry boundary condition was applied on the symmetry plane of the MCHS. A single channel unit was selected as the computational domain, which significantly reduced the calculation costs, while ensuring the accuracy of the results [29,30,31]. Figure 1b illustrates the final numerical simulation calculation domain. The 3D numerical simulations of the heat transfer characteristics of the DL-MCHS with various geometric parameters were examined in detail. Different cross-sectional shapes of microchannels and their dimensional parameters are shown in Figure 2.

In our study, eight different aspect ratios (1.000 to 8.544) of the rectangular MCHSs with the fixed cross-sectional area of 0.36 mm^2^ were chosen for the comparison. The following Table 3 describes their parameters.

The rectangular aspect ratio (*AR*) is defined as:

*AR* = *H_C_*/*W_C_* (1)

The wetted perimeter (*P_h_*) is defined as the perimeter of the conjugate walls of the rectangular channel and is represented as:

(2) $$P_{h}=2W_{C}+2H_{C}$$

The flow and heat transfer performance of the DL-MCHSs with different cross-sectional shapes were studied by analyzing three groups of trapezoidal microchannels and four groups of triangular microchannels. Their cross-sectional areas were the same as those of the rectangular microchannels, and their parameters are listed in Table 4 and Table 5.

## 3. Research Methods

### 3.1. Governing Equations

For the microchannel heat sinks, we made the following assumptions to solve the conjugate heat transfer problem:

(1) This flow is laminar, incompressible, and steady.
(2) The boundary conditions ensure that no slippage occurs.
(3) The radiation heat transfer is negligible, as is gravity.
(4) The solids and liquids have constant properties with the exception of the viscosity of coolant water (the viscosity of water varies with its temperature).
(5) The viscosity dissipation of the water flow is negligible.

A three-dimensional solid-liquid conjugate model was applied considering the assumptions of a steady laminar flow, without giving regard to the gravity and other body forces. The governing equations for the fluid flow and convective heat transfer in microchannels can be depicted as follows:

Mass conservation equation:

(3) $$\nabla \vec{V}=0\;$$

Momentum conservation equation:

(4) $$\rho_{f}\left(\vec{V}\cdot \nabla \vec{V}\right)=-\nabla p+\nabla \cdot \left(\mu_{f}\nabla \vec{V}\right)\;$$

Energy equation:

For the fluid:

(5) $$\rho_{f}c_{p,f}\left(\vec{V}\cdot \nabla T_{f}\right)=k_{f}\nabla^{2}T_{f}\;$$

For the solid:

(6) $$k_{s}\nabla^{2}T=0\;$$

where $\vec{V}$ is the velocity of fluid, *p* refers to the pressure of the fluid regions, *Tf* and *Ts* are the temperature of liquid and solid, respectively. *ρf*, *cp, f*, *μf*, and *kf* are the density, specific heat, dynamic viscosity, and thermal conductivity of water respectively, and *ks* is the thermal conductivity of oxygen-free copper.

The effect of the temperature change on the water viscosity had to be considered in this investigation, which can be calculated from Equation (7), and is fully demonstrated by Kestin et al. [32].

(7) $$\log\left\{\frac{\mu \left(t\right)}{\mu \left(20^{\circ }C\right)}\right\}=\frac{20-t}{t+90}\left\{\begin{array}{c} 1.2378-1.303\times 10^{-3}\left(20-t\right)+3.06\times 10^{-6}{\left(20-t\right)}^{2}\\ +2.55\times 10^{-8}{\left(20-t\right)}^{3} \end{array}\right\}$$

### 3.2. Boundary Conditions

The boundary conditions were set with the working mass of water, and the laminar flow model was chosen. The ambient temperature as well as the fluid temperature was set to a constant 22 °C, and the upper end of the fluid was set to a wall with no viscous resistance. The Reynolds number was set in the range from 200 to 2000 based on the channel size and the dynamic viscosity of the water. The outlet was a pressure outlet boundary condition, and it reached full development at the outlet. The MCHS top surface had a constant heat flow density input (2.7 × 10^6^ W/m^2^). For setting the value of heat flow density of the MCHS, refer to Jia et al. [33]. The inlet velocity was determined by the Reynolds number, and the velocity inlet value was different at the same Reynolds number due to the different hydraulic diameters of the microchannels with different depth-to-width ratios.

The boundary conditions are listed as follows:

No slip boundary conditions are valid on the walls.

A symmetry boundary condition is applied on the symmetry plane of the MCHS.

The inlet of the microchannel:

(8) $$u_{f}=u_{in}=0.34∼5.46\;m/s,T_{f}=T_{\mathrm{in}\;}=295.15\;K\;$$

The outlet of the microchannel:

(9) $$p_{f}=p_{\mathrm{out}\;}=0\;$$

An uniform heat flux is provided to the top surface of the computational domain:

(10) $$q=2.7\times 10^{6}\;W/m^{2}\;$$

The other surfaces are considered as being adiabatic:

(11) $$\frac{\partial T_{s}}{\partial n}=0;\frac{\partial T_{f}}{\partial n}=0\;$$

For the contact surface between the solid and fluid, a coupled boundary condition with no-slip is adopted:

(12) $$\nabla \vec{V}=0;T_{s}=T_{f};-\lambda_{s}\frac{\partial T_{s}}{\partial n}=-\lambda_{f}\frac{\partial T_{f}}{\partial n}\;$$

where λs denotes the solid thermal conductivity.

### 3.3. Numerical Methods and Validations

In this paper, FLUENT 19.2 based on the finite volume method (FVM) was used to analyze the flow and heat transfer characteristics of the MCHS. To solve the fluid-solid coupled heat transfer problem, the same SIMPLEC algorithm was used as in the literature [20,34]. The momentum and energy equations were discretized in second-order upwind schemes. The solutions were considered to have converged when the continuity, velocity, and energy residuals were less than 10^−4^, 10^−4^, and 10^−7^ respectively.

**Figure 3:**
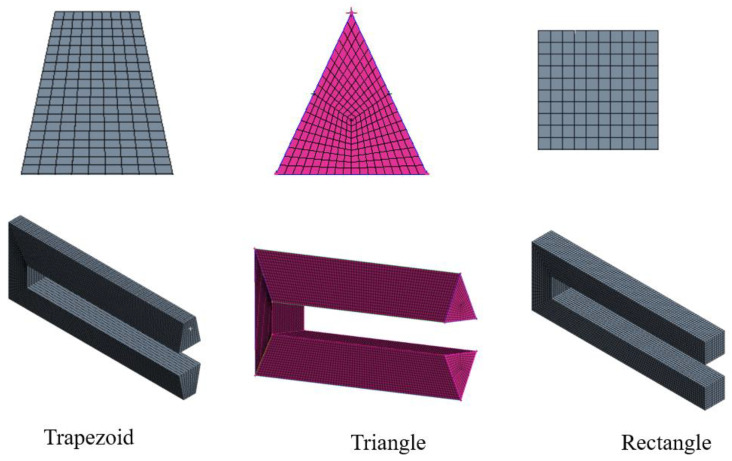
Computational meshes for different cross-section microchannels.

Initially, we examined the mesh independence tests in order to improve the accuracy of our numerical calculations. The mesh independence tests were carried out separately for the MCHSs with different cross-sectional shapes. A series of tests with different numbers of mesh nodes determined the distribution of the hexahedral mesh nodes in the computational domain. ICEM software was used to discretize the spatial region based on high-quality hexahedral meshes, as the entire structure of the microchannel and the computational domain are both hexahedral elements. The boundary layer mesh should be refined in view of the changes in the velocity and pressure at the entrance boundary (to resolve the boundary layer flow, fine meshes were used near the domain walls). Figure 3 shows the computational mesh of the MCHS for three cross-sectional shapes. For the rectangular MCHSs, we used the total pressure drop and thermal resistance of the MCHSs as the target parameters. Table 6 compares the effects of the mesh nodes numbers on the simulation. As can be seen, the total pressure drop increases with the mesh nodes number, while the thermal resistance decreases. However, the total pressure drop and the thermal resistance do not vary obviously when the number of mesh nodes reaches more than 300,641, the variation does not exceed 0.5% when another 350,236, or even more, mesh nodes are added. Therefore, 300,641 nodes were selected as the optimal number of mesh nodes to discretize the computed area. The mesh independence tests for both the trapezoidal and triangular MCHSs were performed using the same method that is described above.

### 3.4. Performance Evaluation Parameters

The Reynolds number of a microchannel can be increased by increasing the inlet velocity of the channel, which can be calculated as follows:

(13) $$Re=\frac{\rho_{f}u_{in}D_{h}}{\mu_{in}}$$

Following equations can be used to calculate *D_h_*, which is the hydraulic diameter.

Rectangular channel:

(14) $$D_{h}=\frac{4A_{C}}{P_{C}}=\frac{2W_{C}H_{C}}{\left(W_{C}+H_{C}\right)}$$

Trapezoidal channel:

(15) $$D_{h}=\frac{4A_{C}}{P_{C}}=\frac{H_{C}\left(W_{b}+W_{t}\right)}{\left(W_{b}+W_{t}\right)+2\sqrt{H_{C}2+{\left(\frac{W_{b}-W_{t}}{2}\right)}^{2}}}$$

Triangle channel:

(16) $$D_{h}=\frac{4A_{C}}{P_{C}}=\frac{2aHc}{\left(a+b+c\right)}$$

Thermal resistance is related to the heat transfer rate of heat sinks, the basic equation for calculating the thermal resistance is:

(17) $$R_{th}=\frac{T_{max}-T_{\mathrm{in}\;}}{Q}=\frac{T_{max}-T_{\mathrm{in}\;}}{A_{b}\cdot q}$$

In this equation, *R_th_* represents the thermal resistance of the microchannel heat sinks, *T_max_* represents the maximum value of the temperature field, and *T_in_* refers to the inlet temperature.

The local heat transfer coefficients [20] and the average Nusselt numbers [35] of microchannels are defined as:

(18) $$h\left(x\right)=\frac{qA_{f}}{A_{b}\left[T_{w}\left(x\right)-T_{f}\left(x\right)\right]}$$

(19) $$Nu\left(x\right)=\frac{h\left(x\right)D_{h}}{k_{f}}$$

*A_f_* represents the total heating area of the high-power laser diode (HPLD) on the microchannel heat sink, and *q* represents the heat flux at the top of the wall. *A_b_* represents the heating area of a single microchannel heat sink. The wall temperature *T_w_* and the bulk fluid temperature *T_f_* are the temperatures along a flow direction, respectively.

The pumping power (*W_pp_*) [20,36] is defined as:

(20) $$W_{pp}=Q_{v}\cdot \Delta P=N\cdot A_{C}\cdot u_{in}\cdot \Delta P$$

There are *N* channels in the microchannel, *A_C_* is the cross-sectional area of each channel, and Δ*p* is the total pressure drop across the microchannel. *W_pp_* denotes the pumping power that is consumed by the microchannel heat sink, *Q_v_* represents the total volume flow rate, and *N* is the total number of channels.

## 4. Result and Discussion

### 4.1. The Influence of Aspect Ratio on Rectangular Microchannel Heat Sinks

**Figure 4:**
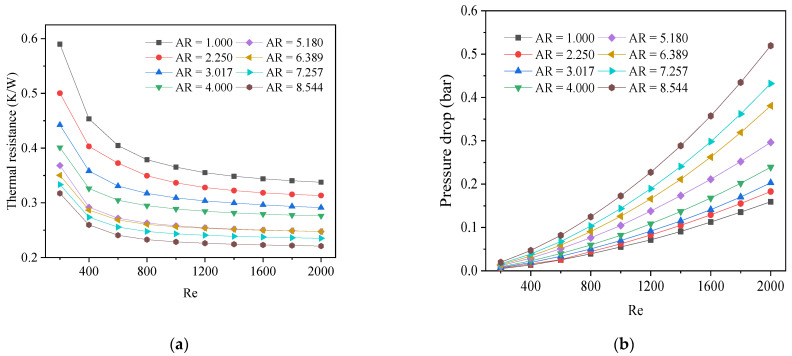
The variation of thermal resistance (**a**) and pressure drop (**b**) for different Reynolds numbers with aspect ratios ranging from 1.000 to 8.544.

Figure 4a illustrates the thermal resistance of the DL-MCHS with various aspect ratios with an increasing Reynolds number, since the cross-sectional area of the microchannel (*A_C_*) was constant at 0.36 mm^2^. When the Reynolds number (*Re*) increased between 200 and 800, the thermal resistance of the heat sinks dropped rapidly with an increasing the inlet velocity. With an *Re* that is above 800, the curve tends to gradually flatten, especially for the high aspect ratios. That is to say that the increase in the velocity no longer improves the heat transfer performance effectively. Further, a higher aspect ratio also results in a lower thermal resistance and a higher pressure drop. When the aspect ratio exceeds five, the curves of the thermal resistance with respect to the Reynolds number were almost unchanged. Further increasing the aspect ratio of the channel is not an effective method to reduce the thermal resistance. On the contrary, the pressure drop increases with the Reynolds number and the aspect ratio (Figure 4b), resulting in a much higher *W_PP_* by the heat sinks (Figure 6a). As the aspect ratio of the rectangular microchannel increased, its hydraulic diameter decreased, while its wet perimeter and convective heat transfer area increased, which improved the heat transfer efficiency and reduced its thermal resistance.

**Figure 5:**
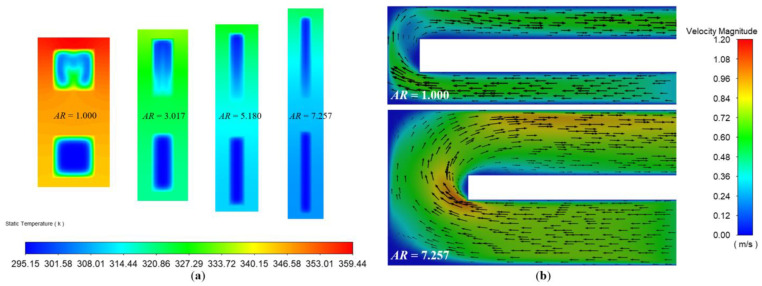
The temperature distribution of the microchannel heat sinks at a distance of 3 mm along the x-direction with different aspect ratios (**a**) (*Re* = 200, Temperature unit: K). The velocity flow diagram of the microchannel heat sinks with different aspect ratios (**b**) (*Re* = 200, Velocity unit: m/s).

The fluid in the boundary layer had a relatively low flow velocity, resulting in a high fluid temperature and a low temperature gradient, which reduced the intensity of the heat transfer [37]. As shown in Figure 5a, in the same Reynolds number condition, the increase in the aspect ratio indicates the thinning of the boundary layer. In particular, the change of the aspect ratio had a greater impact on the low flow rate region at the corner of the microchannel cross-section and at the bottom of the upper microchannel, which played a key role in the improvement of the heat transfer efficiency. According to Figure 5b, by comparing the microchannel velocity flow diagrams for *AR* = 1.000 and *AR* = 7.257 at the same Reynolds number, it could be found that as the microchannel aspect ratio increased, its corresponding low flow velocity region decreased.

**Figure 6:**
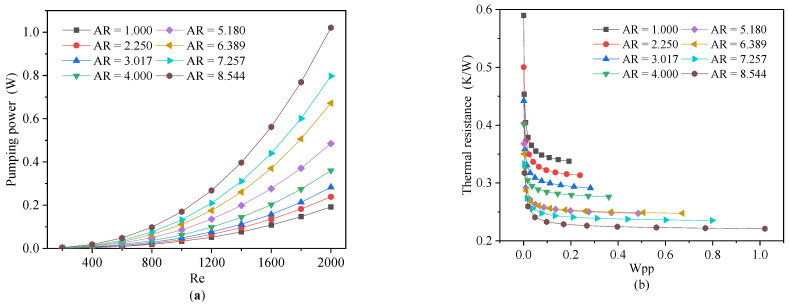
Effect of different aspect ratios on pump power (**a**) and thermal resistance (**b**).

Figure 6b shows the relationship between the thermal resistance and the *W_pp_* that was consumed by heat sinks. As the *W_pp_* increased, for a *W_pp_* that was as low as 0.2 W, thermal resistance dropped sharply for all of the different aspect ratios. However, when the *W_pp_* was more than 0.2 W, the thermal resistance hardly reduced further. With the same *W_pp_* consumption of 0.2 W, the thermal resistance of the rectangular MCHS with an *AR* = 1.000 was 47.58% higher than that of the MCHS with an *AR* = 8.544. Alternatively, for the same *W_pp_*, increasing the *AR* could have a negligible effect on the thermal resistance when the *AR* was greater than five. Consequently, to obtain the best heat transfer performance, the optimal *AR* of the MCHS should be chosen to be around five.

**Figure 7:**
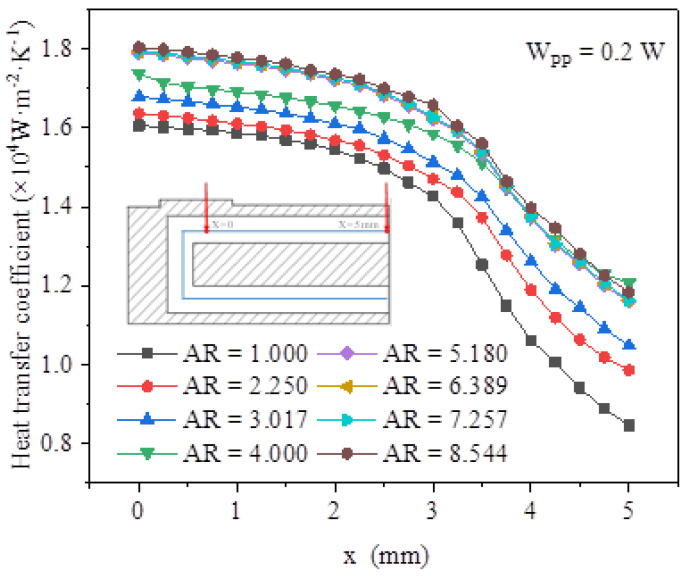
Heat transfer coefficient of rectangular microchannel upper channel with different aspect ratios.

It was crucial to determine the exact correlation of the local heat transfer coefficient (HTC) along the axial distance x based on the numerical results. For the HPLD heat dissipation problems, this was helpful in designing and implementing the MCHSs. As shown in Figure 7, the variation of the HTC in the upper channel of the rectangular MCHS with the rectangular microchannel aspect ratio was investigated at the same *W_pp_* (*W_PP_* = 0.2 W). The HTC decreased continuously along the direction of the water flow. In addition, it was apparent that the HTC increased significantly with an increase in the aspect ratio. However, when the aspect ratio *AR* > 5.180, the values of the HTC were very close. This meant that continuing to increase the aspect ratio on this basis had little effect on improving the HTC. Increasing the aspect ratio of the rectangular microchannel could increase the wet perimeter, i.e., it increased the convective heat transfer area, thereby reducing the heat sink thermal resistance.

**Figure 8:**
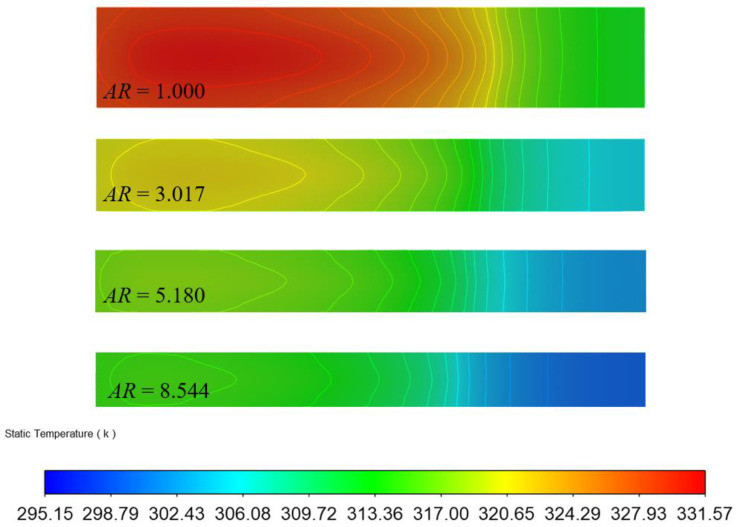
Temperature distribution along x–z of the top of microchannel heat sinks with different aspect ratios (*W_pp_* = 0.2 W, Temperature unit: K).

According to Figure 8, we compared the temperature field distributions of the four groups of the rectangular-topped MCHSs with different aspect ratios along the central axis of the water flow direction. As the result, it was found that the temperature distribution uniformity of the rectangular MCHS was better as the aspect ratio increased. For example, the maximum temperature difference of the rectangular MCHS with an aspect ratio *AR* = 1.000 was as high as 36.43 K, while in comparison, with an *AR* = 8.544, it was was 19.85 K.

### 4.2. Flow and Heat Transfer Characteristics of Triangular and Trapezoidal Microchannels

**Figure 9:**
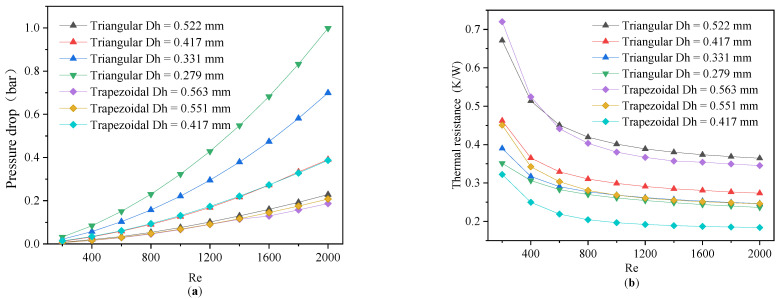
The variation of pressure drop (**a**) and thermal resistance (**b**) with different Reynolds numbers for triangular and trapezoidal microchannel heat sinks.

The different cross-sectional shapes of the DL-MCHSs also had a significant impact on their flow and heat transfer characteristics. The thermal resistance and pressure drop of the triangular and trapezoidal cross-section microchannels of the MCHSs with the increase in the Reynolds number are displayed in Figure 9. With an increasing Reynolds number, the thermal resistance decreased, and the opposite occurred for the pressure drop. The smaller the hydraulic diameter (the height-to-width ratio was increasing) of the microchannel with the same cross-sectional shape was, then the lower the thermal resistance the higher the pressure drop was. As the hydraulic diameter of the microchannel decreased, the wet perimeter and convective heat transfer area were both increased, which improved the heat transfer efficiency and reduced its thermal resistance. Hence, for the MCHSs with trapezoidal and triangular cross-sectional shapes, the narrower the microchannel shape was, the better the heat transfer performance was, however, this occurred at the expense of a high pressure drop.

**Figure 10:**
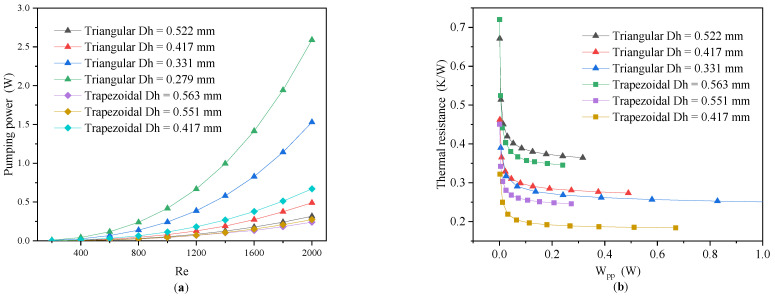
The variation of *W_pp_* (**a**) and thermal resistance (**b**) for triangular and trapezoidal microchannel heat sinks.

Figure 10 shows the variation of the *W_pp_* with the Reynolds numbers (**a**) and the thermal resistance with *W_pp_* (**b**) for the triangular and trapezoidal MCHSs, which were similar to those for the rectangular MCHSs. For the MCHSs with triangular and trapezoidal cross-sectional shapes with a decreasing hydraulic diameter, the *W_pp_* kept increasing at a Reynolds number variation of 200–2000, and the thermal resistance decreased as the hydraulic diameter decreased at the same *W_pp_*.

### 4.3. Comparison of the Flow and Heat Transfer Characteristics of Triangular, Trapezoidal, and Rectangular MCHSs

**Figure 11:**
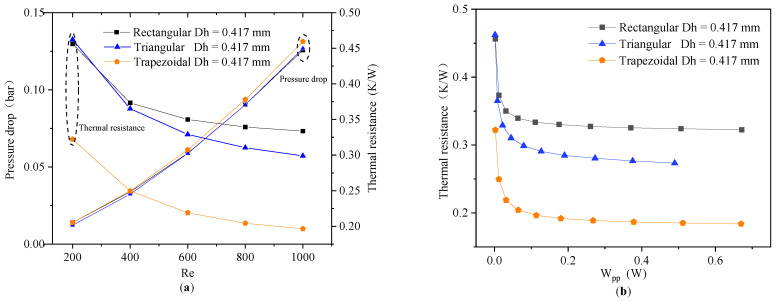
The variation of thermal resistance and pressure drop for different Reynolds numbers (**a**). Thermal resistance under the different *W_pp_* values (**b**).

In this section, we compared the performance of the MCHSs with three cross-sectional shapes of the same cross-sectional area and hydraulic diameter (*A_C_* = 0.36 mm^2^, *D_h_* = 0.417 mm). As shown in Figure 11a, the thermal resistance of the rectangular MCHS was the largest, and that of trapezoidal MCHS was the smallest. As for the pressure drop, the values of the MCHS with rectangular and triangular MCHSs were very close, and the pressure drop of the trapezoidal MCHS was slightly higher. On the other hand, it can be seen from Figure 11b that the thermal resistance of the MCHSs with three cross-sectional shapes tended to decrease as the *W_pp_* increased, and the most rapid reduction in the range of *W_pp_* = 0–0.2 W. Meanwhile, at the same *W_pp_*, the thermal resistance of the rectangular MCHS was the largest; the thermal resistance of the trapezoidal MCHS was the smallest. The thermal resistance of the trapezoidal MCHS was 32.14% and 42.42% lower than that of the triangular and rectangular MCHS, respectively, at the *W_pp_* was 0.2 W.

**Figure 12:**
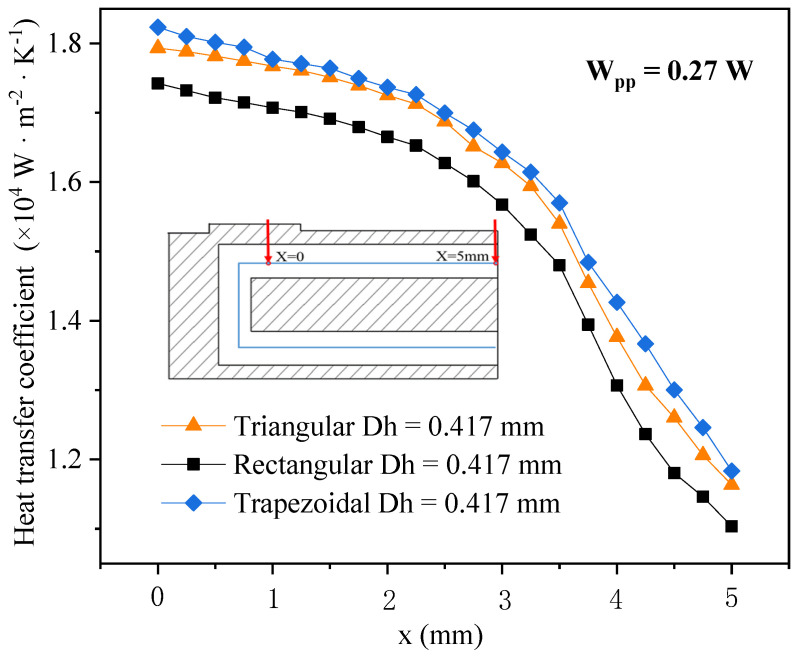
Comparison of local heat transfer coefficients under the same hydraulic diameter (0.417 mm. *W_pp_* = 0.27 W).

**Figure 13:**
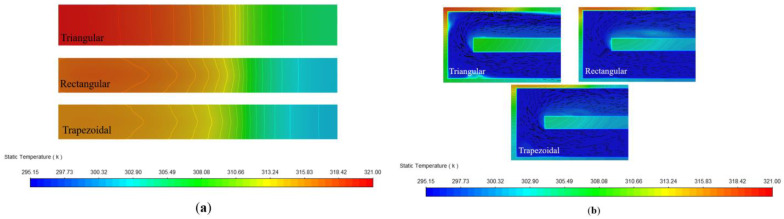
Temperature field along x–z of the top of microchannel heat sinks with different cross-section shapes (*D_h_* = 0.417 mm, *W_pp_* = 0.27 W) (**a**). Temperature field and velocity flow diagram along x–z middle cross-sections of different microchannels (*D_h_* = 0.417 mm, *W_pp_* = 0.27 W) (**b**).

Figure 12 shows that the HTC decreased along the flow direction for the MCHSs with three shapes (*Dh* = 0.417 mm) in the cross-sectional area and the same *W_pp_* consumption value. The HTC of the trapezoidal microchannel was significantly higher than those of rectangular and triangular ones, and the HTC of the rectangles was the smallest. Figure 13a compares the temperature field distribution of the top of MCHSs and the temperature field along the x–z middle cross-section for three cross-sectional shapes (*Dh* = 0.417 mm, *W_pp_* = 0.27 W). It can be seen that the temperature distribution of the trapezoidal MCHS was the most uniform one, and the temperature field gradient of the triangular MCHS was the largest one. According to Figure 13b, by comparing the microchannel velocity flow diagrams for three different cross-sectional MCHSs, at the same *W_pp_*, the triangular MCHS had the most unstable fluid boundary layer and a larger low-flow velocity region in the fluid boundary layer, resulting in a low-temperature gradient.

### 4.4. The Effect of Channel Number on the Performance of Microchannel Heat Sinks

**Figure 14:**
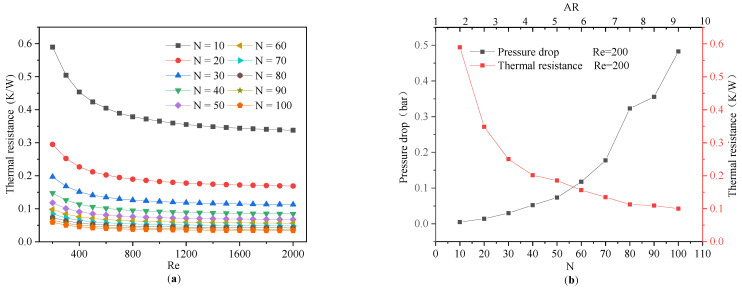
The variation of thermal resistance accompanies the variation of channel number at different Reynolds numbers (**a**). The variation of thermal resistance and pressure drop with change in number of channels at the same Reynolds number (**b**).

The number of channels was also crucial for increasing the performance of an MCHS under the same porosity. The flow area was divided into *N* equal parts in the width direction with the same height, and the total porosity was kept constant to study the impact of *N* on the heat transfer performance of the sink. As shown in Figure 14a, when the *N* increased from 10 to 30, the thermal resistance clearly decreased with the same inlet flow (*Re*). The hydraulic diameter of a single channel was also significantly reduced, while the wet perimeter and the convective heat transfer area of the single microchannel increased. In parallel, the total convective heat transfer surface of the MCHS increased along with an increasing number of channels, which significantly contributed to enhancing the heat transfer properties of the MCHS.

However, the further increase in the value of *N* could not appreciably reducing the thermal resistance as *N* > 30, which was due to the microchannel walls also becoming thinner as *N* increased. For an MCHS with thin channel walls, a high flow resistance resulted in a low heat transfer capacity [38]. Therefore, there would not be further improvement in the heat dissipation once the number of channels were exceeded by a certain number. In addition, the microchannel walls were too thin to be produced during the manufacturing process [39,40].

**Figure 15:**
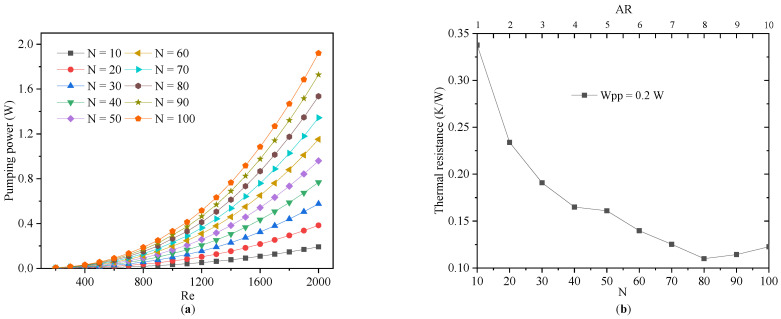
The variation of pump power consumed by MCHSs with different microchannel numbers as Reynolds number increases (**a**). The variation of thermal resistance with the number of channels at the same *W_pp_* (**b**).

For the variation of the pump power that was consumed by the MCHSs with different microchannel numbers, the Re increased, as shown in Figure 15a. In the range of an increasing Reynolds number from 200 to 2000, the *W_pp_* increased significantly, and this growth did not appear to slow even when the *N* was increased to 100. Figure 15b illustrates the relationship between the thermal resistance and *N* under the same *W_pp_* (0.2 W). Initially, at the same pump power, increasing the *N* of the MCHS reduced its thermal resistance, but increasing *N* further lead to an increase in the thermal resistance. In other words, there was an optimal value range for *N* for a particular *W_pp_*. In fact, since the height and porosity of the microchannels were constant, the aspect ratio of the individual channels increased as *N* increased. As discussed in Section 4.1, the large aspect ratio of an MCHS is beneficial to improve the heat transfer efficiency. However, further increasing the *N* of the MCHS will greatly reduce the cross-sectional area of a microchannel and increase the pressure drop. The increased voltage drop resulted in a higher pump power for the MCHS and it offset the gain due to the increased channel count. Kou et al. [15] has also performed similar work.

Therefore, for any given heat sink, the number of channels should have an optimal value, which should be considered in engineering applications. The optimal channel number ranges between 50 and 80 for the MCHSs in this study.

## 5. Conclusions

In this paper, a single variable approach was used to fully research the impact of different parameters (the number of channels, the channel cross-sectional shape, and the aspect ratio) on the temperature distribution, pressure drop, and thermal resistance of the DL-MCHS. Due to a greater wet perimeter and convective heat transfer area, the rectangular DL-MCHS heat transfer performance and pressure drop increased dramatically as the aspect ratio of the channel increased. When the thermal resistance of the DL-MCHSs were compared at the same power consumption, it was discovered that the rectangular DL-MCHS with an aspect ratio of 5.1180–6.389 had the greatest overall performance. Under the same *W_pp_* and the same cross-sectional area and hydraulic diameter (*Ac* = 0.36 mm, *Dh* = 0.417 mm), the thermal resistance of the trapezoidal microchannel heat sink was 32.14% and 42.42% lower than that of the triangular and rectangular ones, respectively. Additionally, when the number of channels inside the DL-MCHS rose, the thermal resistance decreased; nevertheless, this resulted in a greater pressure drop. As a result, there was an ideal range for the channel number, which for the heat sinks that were used in this study was between 50 and 80.

It should be noted that the optimal values of the rectangular aspect ratio and the number of microchannels that were studied in this paper are only valid for the model and aspect ratio that were selected in this paper. It is possible that other conditions may generate changes, thus causing the above optimal values to change as well.

## Figures and Tables

**Table 1:** Thermophysical properties of heat sink substrate materials and cooling fluid.

Materials	Density (kg·m^−3^)	Thermal Conductivity (W·m^−1^·K^−1^)	Specific Heat (J·kg^−1^·K^−1^)
water	1000	0.6	4178
copper	8933	401	385

**Table 2:** Dimensions of the micro-channel heat sink (unit: mm).

*L_y_*	*H_a_*	*L_x_*	*W_C_*	*H_C_*	*W_w_*	*H_top_*	*H_b_*	*L_q_*
10	0.6	6.5	0.6	0.6	0.4	0.15	0.15	3

**Table 3:** Parameters of eight different kinds of rectangular microchannels (*A_C_* = 0.36 mm^2^ unit: mm).

No	*W_C_*	*H_C_*	*D_h_*	*α* = *H_C_*/*W_C_*	*P_h_*
Case1	0.205	1.754	0.368	8.544	3.713
Case2	0.223	1.616	0.392	7.257	3.455
Case3	0.237	1.517	0.410	6.389	3.271
Case4	0.264	1.366	0.442	5.180	2.996
Case5	0.300	1.200	0.480	4.000	2.7
Case6	0.345	1.042	0.519	3.017	2.429
Case7	0.400	0.900	0.554	2.250	2.2
Case8	0.600	0.600	0.600	1.000	1.8

**Table 4:** Parameters of trapezoidal microchannel heat sinks (*A_C_* = 0.36 mm^2^ unit: mm).

No	*W_t_*	*W_b_*	*H_C_*	*D_h_*
Case1	0.142	0.342	1.487	0.416
Case2	0.300	0.500	0.900	0.551
Case3	0.489	0.974	0.492	0.563

**Table 5:** Parameters of triangular microchannel heat sinks (*A_C_* = 0.36 mm^2^ unit: mm).

No	*W_C_*	*H_C_*	*D_h_*
Case1	0.297	2.428	0.279
Case2	0.362	1.989	0.331
Case3	0.493	1.460	0.417
Case4	0.821	0.877	0.522

**Table 6:** Grid independency of hydrothermal results for a rectangular microchannel heat sink.

No	Number of Nodes	*V* (ms^−1^)	*P* (pa)	*R_th_* (kw^−1^)
Case1	177,314	1.6747	5500.557	0.3652
Case2	246,074	1.6747	5926.285	0.3133
Case3	300,641	1.6747	6089.236	0.3083
Case4	350,236	1.6747	6109.457	0.3014

## Data Availability

Not applicable.
